# Drug-Dependent Inhibitory Effects on Corneal Epithelium Structure, Cell Viability, and Corneal Wound Healing by Local Anesthetics

**DOI:** 10.3390/ijms252313074

**Published:** 2024-12-05

**Authors:** Sabine Foja, Joana Heinzelmann, Susanne Hünniger, Anja Viestenz, Christiane Rüger, Arne Viestenz

**Affiliations:** Department of Ophthalmology, University Hospital, Martin-Luther-University Halle-Wittenberg, 06120 Halle (Saale), Germanychristiane.rueger@uk-halle.de (C.R.);

**Keywords:** anesthetics, corneal epithelial cells, porcine corneal organ culture, 3D epithelial culture model

## Abstract

Local anesthetics are commonly used in ophthalmic surgery. However, their use can affect the healing process. This study aimed to investigate the potential impact of anesthetic substances at clinically relevant concentrations and incubation times (3 min), specifically oxybuprocaine (OBPC, 0.4%), lidocaine (LIDO, 2%), and bupivacaine (BUPI, 0.5%), either alone or supplemented with hylase (HYLA, 30 I.E.), on corneal epithelium structure, cell viability, and wound healing. To assess the potential cytotoxicity of these anesthetic substances, viability and colony-forming efficiency (CFE) assays were conducted using the human telomerase-immortalized corneal epithelial (hTCEpi) cell line. Additionally, the toxicity of these substances was evaluated using a 3D human tissue-specific corneal epithelial construct as well as a porcine corneal culture model. The results indicate that OBPC (Novesine^®^ 0.4%) exhibited significant cytotoxicity in 2D and 3D corneal epithelial cell culture models and delayed wound healing in the ex vivo porcine corneal organ culture model. In contrast, LIDO, BUPI, and HYLA were less cytotoxic to corneal cells, with no observed impact on wound healing in the porcine corneal organ culture model. In summary, local anesthetics commonly used in eye surgery are generally considered safe. However, the application of OBPC (Novesine^®^ 0.4%) may delay wound healing.

## 1. Introduction

Local ocular anesthetics are commonly used in ophthalmic surgery to ensure patient comfort during eye procedures. Although local ocular anesthetics are generally well tolerated, they can pose a risk to the physical barrier function of the corneal epithelium, leading to ocular irritation and impaired wound healing due to drug exposure [1,2].

Corneal epithelial cells play a pivotal role in wound healing by migrating across the wound bed and reestablishing the tissue layer to form a protective barrier. Due to the direct contact between this epithelial layer and anesthetic substances after application, possible interferences with wound healing may occur.

Both the literature and occasional observations have indicated that corneal damage and irritation can be observed in some patients after successful surgery. The potential toxicity of these anesthetic substances to ocular surfaces, in both excessive and single-use scenarios, has been a subject of discussion in the literature [3,4]. However, there is a lack of conclusive evidence and consensus on this matter [5].

Therefore, it is essential to conduct clinical observations and basic research to evaluate the potential toxicity of the anesthetics used in surgery. These findings will help to elucidate the potential risks of corneal damage and aid in the development of strategies to optimize wound healing in ophthalmic surgery.

To conduct our investigation, we used the human telomerase-immortalized corneal epithelial (hTCEpi) cell line, an epithelial cell line derived from the limbal region of the cornea. The limbus is the host region of stem cells in the eye and is responsible for epithelial layer regeneration and wound healing. A characteristic feature of these limbal stem cells is their ability to form in vitro compact colonies in culture and to differentiate into three-dimensional (3D) human tissue-specific corneal epithelial constructs [6].

In monolayer culture, we analyzed cell viability and colony-forming efficiency (CFE) after exposure to these anesthetics. Additionally, we used a 3D culture model of tissue-specific differentiated hTCEpi cells to validate the results concerning viability and stratification capacity after exposure to these anesthetic agents. Furthermore, we used an ex vivo injured porcine cornea culture model to examine the influence of anesthetic substances on wound healing.

By employing multiple distinct models, the objective of our study was to analyze the potential toxicity of anesthetic drugs, specifically oxybuprocaine (OBPC), lidocaine (LIDO), bupivacaine (BUPI), and hylase (HYLA), which are commonly used in our hospital. To the best of our knowledge, this is the first study to investigate these anesthetics at routine concentrations, combinations, and incubation times, with a focus on their potential impact on wound healing disturbances.

## 2. Results

### 2.1. Effects of Anesthetic Agents on Corneal Epithelial Cells In Vitro

The in vitro effects of anesthetic agents on corneal epithelial cells were investigated using the human hTCEpi cell line. Compared to that of the control cells, the viability of cells treated with all of the investigated anesthetic substances was reduced. Treatment with OBPC applied via 0.4% Novesine^®^ eye drops had the greatest cytotoxic effect on corneal epithelial cells, resulting in a cell viability of less than 0.1% (*p* < 0.001, 95% confidence interval (CI): −0.10, 0.26).

**Figure 1:**
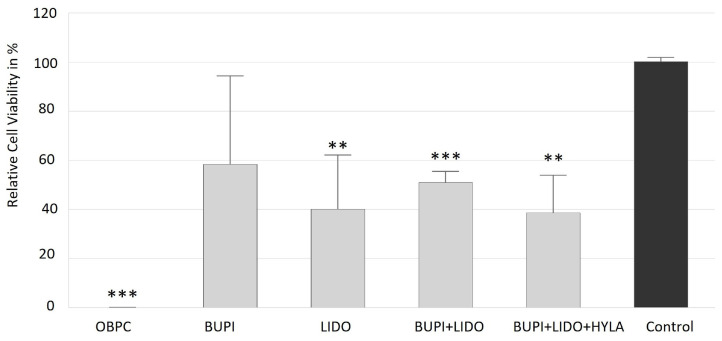
Effects of anesthetics on the viability of hTCEpi cells. Relative viability following incubation with anesthetics for 3 min, washing and further cultivation for 2 d. The median values with 95% CIs represent data from four separate experiments. The *p* values represent data compared to controls (** *p* < 0.01, *** *p* < 0.001). hTCEpi, human telomerase-immortalized corneal epithelial; CIs, confidence intervals; OBPC, oxybuprocaine; BUPI, bupivacaine; LIDO, lidocaine, HYLA, hylase.

BUPI tended to be better tolerated, with a median cell viability of 58.4%. The use of LIDO as an anesthetic resulted in a relative cell viability of 40.12% (*p* = 0.005, 95% CI: 20.10, 66.13). The combined treatment of BUPI+LIDO decreased cell viability to 50.95% (*p* < 0.001, 95% CI: 40.10, 61.39). Moreover, the addition of HYLA (BUPI+LIDO+HYLA) resulted in a relative cell viability of 38.61% (*p* = 0.006, 95% CI: 5.14, 65.46) (Figure 1).

### 2.2. Effects of Anesthetic Substances on the Colony-Forming Efficiency (CFE) of Limbal Stem Cells

hTCEpi cells consist of a mixture of human epithelial and limbal stem cells derived from the limbal region of the cornea. Limbal stem cells play a crucial role in maintaining corneal clarity and are involved in the wound healing process. A characteristic feature of these stem cells in culture is their ability to form tight, compact colonies. Consequently, the number of colony-forming cells is used as an indicator of the presence of intact stem cells in the culture. We therefore analyzed the CFE of hTCEpi cells after treatment with anesthetic substances for 3 min.

**Figure 2:**
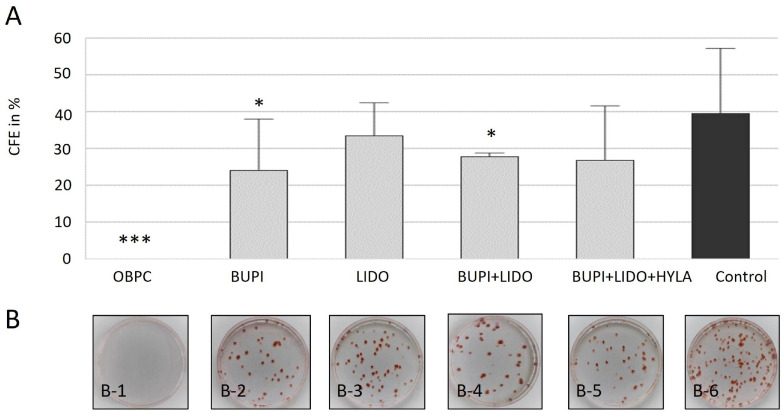
Effects of anesthetics on colony-forming ability of hTCEpi cells. (**A**) Calculation of CFE. The median values with 95% CIs represent data from four separate experiments. The *p* values represent data compared to controls (* *p* < 0.05, *** *p* < 0.001). (**B**) The images depict the colony formation assay results obtained 10 d after treatment, with 250 cells seeded per dish. On day 2, the cells were treated with anesthetic agents for 3 min. The dishes were fixed on day 12 of culture and stained with Congo Red. The results show the effects of treatment with OBPC (B-1), BUPI (B-2), LIDO (B-3), BUPI+LIDO (B-4), BUPI+LIDO+HYLA (B-5), and the untreated control group (B-6). hTCEpi, human telomerase-immortalized corneal epithelial; CFE, colony-forming efficiency; CIs, confidence intervals; OBPC, oxybuprocaine; BUPI, bupivacaine; LIDO, lidocaine, HYLA, hylase.

As shown in Figure 2A, the control group achieved a CFE of 41.3%. Following treatment with the anesthetic substance OBPC (Novesine^®^ 0.4%), no colonies were detected (*p* < 0.001) (Figure 2A,B). Thus, in four independent experiments, no surviving stem cells were observed after incubation with 0.4% Novesine^®^. Cells incubated with BUPI or LIDO alone had a CFE of 23.9% (*p* = 0.047, 95% CI: 0.33, 34.39) and 33.4%, respectively. The combination of BUPI+LIDO led to a CFE of 27.8% (*p* = 0.039, 95% CI: 0.95, 29.97). Finally, treatment with BUPI+LIDO+HYLA resulted in a CFE of 26.8% (*p* = 0.088, 95% CI: −2.65, 31.18).

### 2.3. Impact of Anesthetics on the Capacity of Cells to Form the Human Tissue-Specific Corneal Epithelial Layer

Tissue-specific corneal epithelial constructs can be generated by differentiating hTCEpi cells using high-calcium culture medium and airlifting. Therefore, differentiated hTCEpi cells serve as a corneal epithelium culture model and can provide insights into the impact of anesthetic substances on the differentiation capacity of these cells.

To investigate the possible influence of anesthetic agents on the development of the corneal epithelium layer, hTCEpi cells (2D culture) were treated with antiseptic substances for 3 min and cultured for an additional 48 h. Following this treatment, the cells were subjected to differentiation for 21 d.

At the end of the 21 d differentiation period, treatment with BUPI or LIDO, either alone or in combination with and without HYLA, did not impair the cells’ ability to form the corneal epithelium layer. These differentiated constructs retained the typical characteristics of the human corneal epithelium, including basal cells, wing cells, and surface cells.

**Figure 3:**
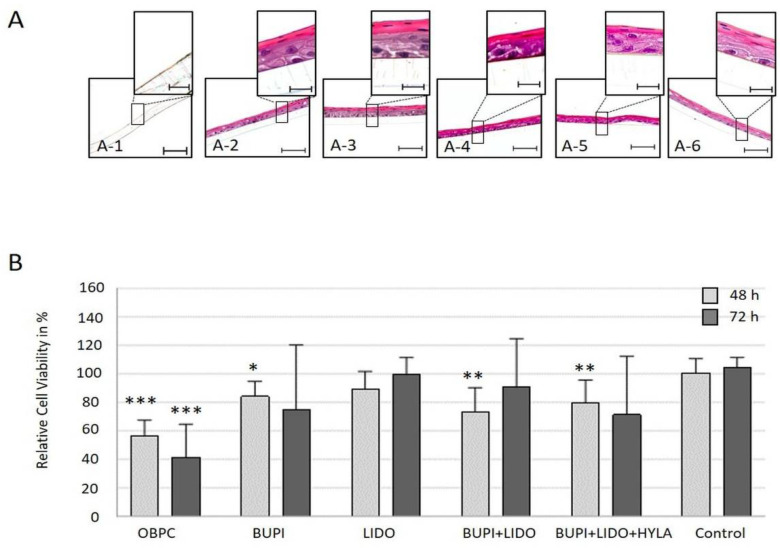
Functionality and viability of tissue-specific corneal epithelial constructs. (**A**) Immunohistochemical detection of the differentiation potential of hTCEpi cells, 21 d after treatment with an anesthetic substance (HE-stained). hTCEpi cell cultures were treated with OBPC (A-1), BUPI (A-2), LIDO (A-3), BUPI+LIDO (A-4), or BUPI+LIDO+HYLA (A-5). The control was cultured in medium (A-6). The representative images represent the results of four separate experiments, each performed in triplicate. The scale bars represent 50 µm (bottom) and 10 µm (top). (**B**) Relative viability of cells 48 h and 72 h after exposure of the tissue-specific corneal epithelial construct to anesthetic substances. The median values with 95% CIs represent data from four separate experiments, each performed in triplicate. The *p* values represent data compared to controls (* *p* < 0.05, ** *p* < 0.01, *** *p* < 0.001). hTCEpi, human telomerase-immortalized corneal epithelial; CIs, confidence intervals; OBPC, oxybuprocaine; BUPI, bupivacaine; LIDO, lidocaine, HYLA, hylase.

However, the application of OBPC to the 2D cultured hTCEpi cell line led to significant cell loss. Consequently, a sufficient number of cells for the differentiation approach was unavailable (Figure 3A).

### 2.4. Effects of Anesthetics on the Viability of Cells in the Human Tissue-Specific Corneal Epithelium Construct

To assess the influence of anesthetic substances on the viability of cells in the tissue-specific corneal epithelium construct, a 21-day differentiation approach was used. The tissue-specific corneal epithelium construct (3D culture model) was incubated with anesthetic substances for 3 min, followed by washing and further incubation for 48 h and 72 h, respectively. Following this treatment, the viability of the cells in the tissue-specific corneal epithelial constructs was analyzed, and the results are shown in Figure 3B.

Treatment with OBPC significantly reduced the median viability of cells in the tissue-specific corneal epithelial construct to 56.2% (*p* < 0.001, 95% CI: 41.38, 71.69) and 41.7% (*p* < 0.001, 95% CI: 41.38, 71.69) after 48 h and 72 h, respectively. After the application of BUPI, the cell viability significantly decreased to 84.2% (*p* = 0.012, 95% CI: 62.75, 94.95) after 48 h and to 75.2% after 72 h (*p* = 0.089, 95% CI: 57.08, 102.59). After incubation with LIDO, the relative cell viability decreased to 89.2% and 99.4% after 48 h and 72 h, respectively. The combination of BUPI+LIDO led to a slight reduction of viability to 73.4% (*p* = 0.003, 95% CI: 52.89, 88.91) after 48 h of culture, and 90.5% after 72 h of culture post-treatment. Finally, the combined application of BUPI+LIDO+HYLA resulted in a cell viability of 79.7% (*p* = 0.006, 95% CI: 65.00, 93.21) after 48 h of culture and 71.5% after 72 h (*p* = 0.057, 95% CI 59.70, 100.61) of culture post-treatment.

### 2.5. Wound Healing Capacity of Ex Vivo Porcine Corneal Cells After Anesthetic Treatment

**Figure 4:**
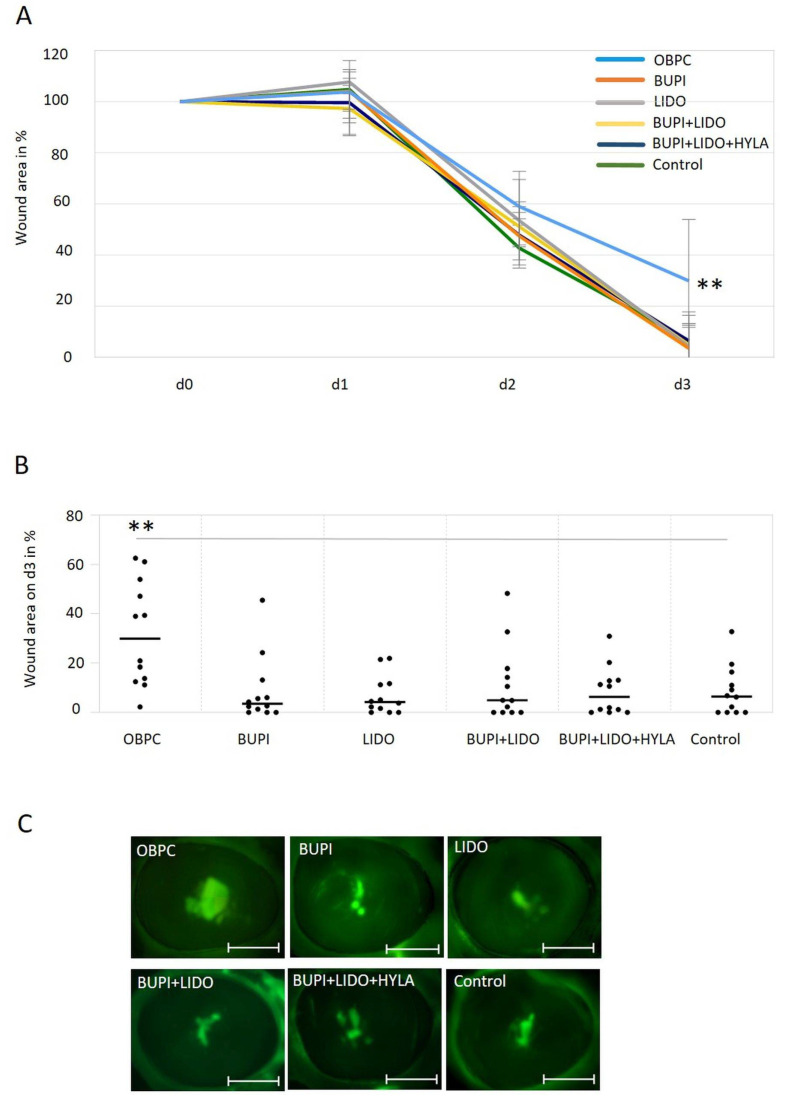
Impact of anesthetics on wound healing in an ex vivo porcine corneal organ culture model. (**A**) Changes in the wound area of porcine corneas after treatment with anesthetic substances for 3 min and culture for up to 3 d. The data represent the results of three independent experiments with 12 eyes per treatment group. The median values with 95% CIs are presented. *P* values represent data compared to controls (** *p* < 0.01). (**B**) The percentage of the remaining wound area on day 3 in porcine corneas following a 3 min treatment with different anesthetics, with the wound area on day 0 set as 100%. The line represents the median, and each point indicates the percent wound area for each cornea after three separate experiments (n = 12 per treatment group, ** *p* < 0.01). The gray line marks the 2 groups with significantly different wound healing results (comparison control—OBPC). (**C**) Representative examples of fluorescein-stained wounds in porcine corneas on day 3. Scale bars indicate 5 mm. CIs, confidence intervals; OBPC, oxybuprocaine; BUPI, bupivacaine; LIDO, lidocaine, HYLA, hylase.

Following treatment of the porcine corneal epithelium wound area with various anesthetic substances, a detrimental impact of OBPC on the wound healing process was demonstrated. On day 3 of culture, the median residual wound area was significantly greater (29.86%) in the group treated with OBPC than in the untreated control group (6.51%) (*p* < 0.01, 95% CI: 9.1, 37.15) (Figure 4A). The distribution of residual wound areas, 3 d post-treatment is illustrated in Figure 4B.

No significant differences were observed between the groups treated with the other anesthetics and the untreated control group (Figure 4A–C).

## 3. Discussion

For more than a century, topical ocular anesthetics have been used for effective pain control. These substances are in direct contact with the corneal epithelium, a cell layer that is essential for promoting wound healing, maintaining a refractive surface to see properly, and acting as a barrier to protect ocular tissue.

While anesthetics are generally considered safe, local anesthetic agents carry potential risks of irritation of the ocular surface, disruption of tear film stability, impairment of epithelial cell motility, as well as disruption of the function of epithelial and limbal stem cells, which may occasionally result in delayed wound healing [1,2]. Furthermore, there is a lack of consensus in the literature regarding the recommended type of anesthesia, the optimal concentration, and the optimal treatment duration for eye surgery.

Further, prolonged or excessive use of local anesthetics may result in delayed corneal re-epithelialization, long-term epithelial cell dysfunction, and impaired epithelial cell motility and wound healing [7,8]. Moreover, even a single application of topical anesthetics can lead to harmful changes in the corneal epithelium [4,9].

Therefore, the primary objective of this study was to examine the potential cytotoxic effects of anesthetic substances on corneal epithelium, which could be a possible source of occasional corneal damage and irritation symptoms after successful surgery. Because of that, we investigated commonly used anesthetics for eye surgery, particularly 0.4% OBPC, 0.5% BUPI, and 2% LIDO, as well as a combination of BUPI+LIDO, and BUPI+LIDO+HYLA in a clinically relevant contact time of 3 min.

The diversity of the selected anesthetics is well justified, as each agent offers distinct characteristics suited to different clinical needs. OBPC, an ester-type anesthetic, acts within seconds when applied topically but has a short duration of 10–15 min. LIDO has a rapid onset within 1 min and lasts 1–3 h. BUPI has a slower onset of around 10 min but offers a much longer duration of 6–8 h. Therefore, for rapid onset and extended anesthesia, a combination with LIDO is often used [1,10,11].

Acknowledging that the results obtained from monolayer cell culture experiments may differ due to the microenvironmental differences from those obtained using 3D tissue-specific models [1], we complemented our investigations with 3D models to enhance their relevance for practical applications.

OBPC was investigated in our study using Novesine^®^. Our findings revealed pronounced toxicity of OBPC in both monolayer-cultured corneal epithelial cells and 3D tissue-specific corneal epithelial constructs. Additionally, OBPC significantly impaired the wound healing process in an ex vivo cultured porcine cornea model. Previous studies have confirmed the dose- (from 0.025% to 0.4%) and time-dependent (up to 28 h) cytotoxicity of OBPC on 2D cultures of human corneal epithelial cells, showing effects such as cell cycle arrest, mitochondria-dependent apoptosis, and the induction of plasma membrane permeability [1]. These observations are consistent with clinical findings in which patients exhibited keratitis and corneal peripheral infiltration after multiple topical applications of 0.4% Novesine^®^ for up to 4 d [12]. Nevertheless, in a multicenter prospective study of 152 patients with severe to complete limbal stem cell deficiency, autologous limbal stem cells were injected under 0.4% OBPC anesthesia, resulting in a success rate of 66% [13].

BUPI is known for its prolonged duration of action compared to that of LIDO [11], and it is often used alone or combined with LIDO or LIDO+HYLA due to the faster onset of the mixture compared to the agent alone [14]. Our study revealed that BUPI alone or in combination with LIDO and HYLA did not significantly negatively affect the viability, differentiation, or wound healing process of corneal epithelial cells.

Therefore, to our knowledge, for the first time, we have shown that 0.5% BUPI, alone and in combination with LIDO and HYLA, is safe to use and does not impair the wound healing processes in the cornea.

LIDO is frequently used as a local anesthetic, either alone or in combination, typically at concentrations ranging from 0.5 to 2.0% [8,11]. In 2D monolayer culture of corneal epithelial cells, our observations revealed a substantial reduction of viability of corneal epithelial cells by more than 50% after application of 2% LIDO for 3 min. Hirata et al. have supported these results. They have shown that LIDO reduces cell proliferation by inhibiting the tyrosine kinase activity of the epidermal growth factor receptor [7].

However, when LIDO was applied to in vitro and ex vivo 3D culture models, we found no significant alterations in the viability, cell differentiation, or wound healing ability of corneal epithelial cells. The available literature provides only limited data on the effect of LIDO on ocular wound healing. Notably, Bisla et al. [8] reported a dose-dependent effect of LIDO on wound healing in the rabbit eye. However, it is important to note that their investigations used lower concentrations (up to 0.1%) and significantly longer incubation times (up to 60 h), whereas our study focused on a brief 3 min exposure aligning with operative routines.

Based on our findings, we concluded that LIDO is not responsible for wound healing disturbances in our study.

HYLA has been utilized as an adjuvant in the BUPI+LIDO combination for retrobulbar and peribulbar blocks to increase the success of regional anesthesia for the eye [15]. However, in cases of hypersensitivity reactions, HYLA may be associated with postoperative periorbital inflammation symptoms appearing up to 3 d after surgery [16]. In our study, we found that coadministration of HYLA did not significantly influence cell viability, cell functionality, or wound healing capacity.

In conclusion, based on our findings, OBPC (0.4% Novesine^®^) may not be a recommended option due to its potential toxicity to ocular cells and its negative impact on corneal wound healing. In contrast, the use of LIDO or BUPI, alone or in combination with or without HYLA, have shown no negative side effects in terms of cell viability, functionality, and the wound healing process.

This study has some limitations. Most notably, it is only the analysis of the commercially available anesthetic preparations. Further studies are recommended to distinguish the potential effects of the drug from those of the excipients. In addition, we tested the routinely used incubation time of three minutes. It is important to clarify whether extended incubation times, as may be practiced in other hospitals, could lead to additional cytotoxic effects.

## 4. Materials and Methods

### 4.1. Anesthetics

The anesthetics used in this study were oxybuprocaine hydrochloride (OBPC, Novesine^®^ 0.4%, OmniVision GmbH, Puchheim, Germany), bupivacaine hydrochloride (BUPI, Carbostesin^®^ 0.5%, Aspen GmbH, Munich, Germany), lidocaine hydrochloride (LIDO, Xylocitin-Ioc^®^ 2%, mibe GmbH, Brehna, Germany), and hyaluronidase (Hylase^®^ “Dessau” 300 I.E., Remser Pharma GmbH, Greifswald, Germany). Bupivacaine and lidocaine (BUPI+LIDO) were combined at a ratio of 1:1.

Hyaluronidase, also known as hylase (HYLA), was dissolved in LIDO and used at a concentration of 30 I.E. in the combination treatment of BUPI and LIDO (BUPI+LIDO+HYLA).

For all anesthetics, the treatment duration was generally 3 min, followed by 3 washes with PBS for 1 min each.

### 4.2. Cell Culture

The hTCEpi cell line, derived from the limbal region of corneal tissue, was purchased under Order No. CHT-045-0237 (Everyte, Vienna, Austria). This cell line was chosen for this study to model the corneal epithelium, a cell layer that directly interacts with anesthetics during surgical procedures and plays a key role in promoting wound healing. These cells express limbal stem cell markers and can be differentiated into a 3D tissue-specific corneal epithelial cell construct [6]. The hTCEpi cells were maintained in Keratinocyte Growth Medium (KGM)-2 supplemented with KGM-2 SingleQuot Kit supplements (Lonza, Basel, Switzerland). The cell line was cultured in a 5% CO_2_ humidified atmosphere at 37 °C and passaged every 3 d to 5 d following the standard operating procedure described by the manufacturer’s instructions. The cell lines were authenticated to ensure their identity and purity.

### 4.3. CFE Assay

For the colony-forming assay, 250 cells were plated on 100 × 20 mm cell culture dishes (Sarstedt AG, Nuremberg, Germany) and cultured in complete culture media. On day 2, the cells were treated with anesthetic agents for 3 min and cultured for an additional 12 d. Untreated cells were used as controls. The cell colonies were fixed with 4% paraformaldehyde (PFA, Thermo Fisher Scientific, Carlsbad, CA, USA) for 30 min and rinsed with PBS. Prior to colony counting, the cells were stained with Congo Red dye for 15 min at room temperature (Carl Roth GmbH, Karlsruhe, Germany). The CFE was calculated using the following equation: CFE = (number of colonies per plate/number of seeded cells) × 100. This experiment was performed using four different biological replicates.

### 4.4. Differentiation into Tissue-Specific Corneal Epithelial Constructs

To induce the differentiation of hTCEpi cells to generate a tissue-specific corneal epithelial construct, a total of 1.7 × 10^4^ hTCEpi cells were cultured in the upper chamber of a Transwell system with ThinCerts culture inserts (24-well, 0.4 µm pore size, Greiner Bio-One GmbH, Kremsmünster, Austria) containing KGM-2 culture medium including 1.15 mM Ca^2+^ in both chambers. The medium was changed daily for 7 d. On day 8, the medium was removed from the upper chamber, to create an airlifting cell culture. For an additional 14 d, the cells were cultured by renewing the medium in the lower part of the system daily. The cells were maintained in a humidified atmosphere at 37 °C with 5% CO_2_.

### 4.5. Cell Viability

To analyze the viability of cells in the monolayer culture model, hTCEpi cells were seeded at a density of 5 × 10^3^ cells/well in 24-well plates and cultured for 48 h. Subsequently, the cells were incubated with anesthetics for 3 min. The cells were then cultured for an additional 48 h. The medium control group consisted of cells that did not receive anesthetic treatment. Cell viability was determined using the CellTiter-Glo assay (Promega, Walldorf, Germany) following the manufacturer’s instructions. Briefly, the CellTiter-Glo reagent was equilibrated to room temperature. After adding the CellTiter-Glo reagent to the cells, the plates were vigorously mixed for 5 min. Following an additional incubation period of 20 min, luminescence was measured using a microplate reader (Infinite 200 reader, Tecan, Nänikon, Switzerland).

Viability analysis of the 3D tissue-specific corneal epithelial constructs was performed after incubation with anesthetics for 3 min, followed by further culture for 2 d and 3 d. The cells were incubated with trypsin for 25 min at 37 °C to detach them from the ThinCerts. The reaction was then inactivated using a trypsin inhibitor at a 1:1 ratio. After resuspending of the cell-trypsin and trypsin inhibitor suspension, the cells were transferred into reaction vials. Following washing of the insert membrane with medium, Celltiter-Glo was added at a 1:1 ratio. Subsequently, cell lysis of the 3D construct was achieved by shaking at 2000 rpm for 6 min at room temperature using a ThermoMixer (Eppendorf, Hamburg, Germany), followed by an ultrasonic bath for 5 min (EMAG AG, Mörfelden-Walldorf, Germany). After an additional 20 min of incubation at room temperature, the luminescence was measured using a microplate reader. All experiments were performed in biological triplicates and repeated 4 times (n = 12). The results are expressed as the percentage of cell viability compared to the non-treated control.

### 4.6. Immunohistochemistry and Microscopy

Inserts containing the tissue-specific corneal epithelial constructs were fixed in 4% PFA for 48 h, followed by storage in 70% ethanol. The tissues were embedded in paraffin using a Histokinette tissue processor (Leica, Wetzlar, Germany). Tissues were cut into 4 µm sections using a rotary microtome (Leica, Wetzlar, Germany). The paraffin-embedded sections were incubated at 60 °C for 2 h and subsequently dehydrated with Roti^®^Histol (Carl Roth, Karlsruhe, Germany) and decreasing ethanol gradient series, followed by the addition of distilled water. Sections were stained using hematoxylin and eosin (H&E) at the Institute of Pathology, Martin-Luther University, Halle (Saale).

### 4.7. Ex Vivo Culture of Porcine Corneas

Fresh porcine eyes were obtained from a slaughterhouse (Tönnies, Weissenfels, Germany). The eyes were prepared following a modified protocol based on Castro et al. [17]. Briefly, the eyes were collected within 3 h of extraction and transported on ice in PBS supplemented with 1× antibiotic–antimycotic solution (ABAM, ThermoFisher Scientific, Karlsruhe, Germany) to the laboratory for immediate processing. Excess muscle and connective tissue were removed, and the bulbi were washed in sterile PBS. Subsequently, the bulbi were decontaminated with a 2% iodine solution for 2 min and washed twice again with PBS.

Corneal wounds were created by removing the corneal epithelium using a 7 mm trephine and a hockey knife. The corneas were separated from the globes, leaving a small rim of scleral tissue. To maintain the native corneal structure during culture, the endothelial side of the corneas was filled with sterile 1% agar in Culture Medium II (PAN Biotech, Aidenbach, Germany) and 1 mg/mL bovine collagen (CellSystems, Troisdorf, Germany), and carefully flipped over for liquid–air interface culture. Subsequently, the corneas were treated with anesthetic substances for 3 min and washed three times with PBS. Untreated control corneas were used as the reference group.

The corneas were cultured in 6-well plates containing Culture Medium II supplemented with 2.5× ABAM and 1.5% gentamycin (50 mg/mL, Th. Geyer, Renningen, Germany) under airlifting conditions. The medium was changed daily, and the cornea surfaces were moistened with media.

To evaluate the effect of different treatments on epithelial wound healing, fluorescein staining of the corneas was performed using a 0.08 ng/mL fluorescein solution (Novartis, Nuremberg, Germany) diluted in balanced salt solution (BSS). The wound healing area was determined by capturing fluorescence images of the cornea surface using an Axio Zoom V16 microscope (Carl Zeiss, Oberkochen, Germany) and calculating the stained area with the microscopy software Zen Blue (Zen 2.5, Version 2.5.75.0, Carl Zeiss, Oberkochen, Germany).

After 3 d of cultivation, the corneas were fixed in 4% PFA for 48 h for H&E staining. The experiments were repeated three times, with four corneas in each group (n = 12).

### 4.8. Statistical Analyses

The statistical analyses were performed to calculate the relative cell viability of the corneal epithelial construct after treatment with the anesthetic substances, as well as to determine the degree of wound healing in pig corneas. A *t*-test, in conjunction with Levene’s test, was used for these analyses. The levels of significance were set at * (*p* < 0.05), ** (*p* < 0.01), and *** (*p* < 0.001). The statistical analyses were performed using IBM SPSS Statistics (SPSS, Inc., Chicago, IL, USA, version 28).

## Data Availability

Data is contained within the article.
